# A program evaluation of Kids2Hear, a student-run hearing screening program for school children

**DOI:** 10.1186/s40463-016-0159-x

**Published:** 2016-09-26

**Authors:** Tina Hu, Katherine Stead, Terence Fu, Blake Papsin

**Affiliations:** 1 Faculty of Medicine, University of Toronto, Toronto, Ontario Canada; 2 Department of Otolaryngology Head and Neck Surgery, Hospital for Sick Children, 555 University Avenue, Toronto, Ontario M5G 1X8 Canada

**Keywords:** Pediatrics, ENT, Family practice/general practice/primary care, Medical education

## Abstract

**Background:**

Hearing deficits in children are demonstrably negatively associated with language acquisition and cognition. Although universal neonatal hearing screening exists, it is not offered equally across Canada. Additionally, children emigrating from other countries are often not assessed. The objective of this study is to evaluate Kids2Hear, a free hearing screening program run by medical students at elementary schools, and to determine the rate of hearing deficits that were identified and referred for evaluation.

**Methods:**

Retrospective analysis of screening program data from 228 participants seen at three inner-city elementary schools over six months.

**Results:**

In our sample, the mean age was 5.8 ± 1.0 years with 48 % males. Approximately 21 participants (9.3 %) were screened positive for a hearing deficit and required referral for supplementary audiological evaluation. About 44 participants (19.3 %) were referred to a family physician for otoscopic abnormalities. Females were significantly more likely to be identified for both hearing deficits and otoscopic abnormalities.

**Conclusions:**

Hearing deficits and otoscopic abnormalities are common among young children. Female children may be at higher risk for developing hearing issues or otoscopic abnormalities compared to males. Additional research is needed to determine the effectiveness of hearing screening programs.

## Background

Failure to identify children with congenital or acquired hearing loss can result in lifelong consequences including deficits in speech and language acquisition, poor academic performance, poor cognition [1, 2], poor psychosocial skills [3, 4], underemployment, and psychological distress [5]. For example, a delay in diagnosis and intervention, and the severity of the hearing deficit have been shown to be directly proportional to the effect on the child’s future linguistic skills [1]. Identifying hearing deficits at a young age can help prevent or reduce many of these adverse consequences by allowing early rehabilitation.

Although most children with congenital hearing loss are identified by newborn hearing screening, some acquired or progressive hearing deficits may not be apparent until later in childhood [6]. Acquired causes of hearing loss may include infectious causes such as otitis media, high noise levels, and ototoxic drugs [3, 7]. In addition, only five provinces in Canada (British Columbia, Ontario, Nova Scotia, Prince Edward Island, and New Brunswick) offer full or partial newborn hearing screenings. Early Hearing Detection and Intervention (EHDI) programs in Canada are designed to be more comprehensive than a universal newborn hearing screening program and include all aspects of screening for hearing loss in infants, identifying hearing loss in those referred from screening, and intervention services in those with hearing deficits. Only one Canadian province (British Columbia) has a comprehensive EHDI program with strong protocols and standards in place [8]. These newborn and early hearing screening programs are not consistently offered in other countries. As a result, children who have immigrated to Canada are often not assessed.

Kids2Hear is a primary preventative health initiative run by medical students at the University of Toronto (Toronto, Ontario, Canada). In this program, trained medical students screen kindergarten students for hearing deficits and otoscopic abnormalities under the supervision of licensed audiologists and otolaryngologists from the Hospital for Sick Children (HSC; Toronto, Ontario, Canada). This free program is geared towards inner-city school students that reside in lower socio-economic areas as well as schools where there are a large proportion of children who have recently immigrated to Canada. Children who are identified with a hearing deficit through audiometry are referred to a pediatric audiology and otolaryngology clinic while children with otoscopic abnormalities are referred to a local family physician.

The goal of this study was to evaluate a free hearing screening program offered at elementary schools and determine the rates of hearing deficits that were identified and referred for medical evaluation.

## Methods

This study was granted program evaluation status by the University of Toronto Research Ethics Board. The study represents data collected over 6 months (October 2014 to April 2015) through the Kids2Hear program, a student-run initiative at the University of Toronto. As part of the program, medical students were trained by HSC staff in audiological screening and otoscopy. Otoscopy training involved both teaching sessions with OtoSim (an ear training and simulation system) and practice with live subjects (medical student peers).

Students from three Toronto elementary schools participated in the Kids2Hear program for the 2014–2015 school year. Consent forms were sent several weeks in advance to the school’s administration to distribute to all students in classrooms whose schedules permitted them to participate. Only those students presenting on the screening day with a consent form completed by their parent/guardian were eligible for hearing screening.

Audiological screening consisted of a pure tone screen (30 dB HL) bilaterally assessing frequencies of 1000 Hz, 2000 Hz, and 4000 Hz. Otoscopic inspection was performed bilaterally. The University of Toronto medical students worked side-by-side with professionals from HSC who volunteered to help with the screening sessions. Audiologists supervised medical students conducting audiological screening in a one-to-one ratio, and a staff or resident otolaryngologist confirmed each of the medical student’s otoscopic inspection findings. If a child was identified with deficits or otosopic abnormalities during the screening, Kids2Hear coordinated a referral to 1) the pediatric audiology and otolaryngology clinic at HSC for audiological deficits or 2) a local family physician for children with only otoscopic abnormalities.

Data were analyzed using SPSS version 20 (IBM, Armonk, NY). Descriptive analyses using chi square analyses were used to examine characteristics of the overall sample and assess for gender effect. Significance was assigned at *p* < 0.05.

## Results

The overall cohort consisted of 228 participants (48 % male) from three elementary schools in Toronto. Demographic information about the schools and students screened in the Kids2Hear program by school is shown in Table 1; school demographic information was obtained from the Toronto District School Board. [9] At all three schools, a large proportion of the children (43–78 %) had a primary language other than English. Approximately 3 to 6 % of children had lived in Canada for less than 2 years, and 3 to 6 % had lived in Canada for 3 to 5 years. Participants ranged in age from 5 to 9 years (*M* = 5.8, *SD* = 1.0) and from kindergarten to grade 3. Table 2 shows the area (ward) socioeconomic information data for each school. The school wards participating in this program had a lower household annual income compared to the average in Toronto (Ward A [Schools 1 and 3], $67,305; Ward B [School 2], $60,555; Toronto, $87,038).

**Table 1:** School and demographic information

	School 1	School 2	School 3
Total number of students	382	324	257
Gender	55 % male	48 % male	49 % male
Primary language other than English	43 %	78 %	44 %
Students living in Canada for 2 years or less	3 %	4 %	6 %
Students living in Canada for 3–5 years	6 %	3 %	5 %
Total number of students screened in Kids2Hear program	47	94	87
Gender of students screened in Kids2Hear program	47 % male	43 % male	55 % male
Audiological evaluation needed	15 %	12 %	3 %
Family medicine referral needed	13 %	7 %	36 %

Information obtained from the Toronto District School Board school profiles [9]

**Table 2:** Demographic information for areas surrounding schools

	Ward A (School 1 and 3)	Ward B (School 2)	Toronto
Annual household income	$67,305	$60, 550	$87,038
Proportion with household income less than $20,000	15 %	23 %	15 %
Incidence of low income households	23 %	20 %	19 %
Born outside of Canada	41 %	59 %	51 %
Unemployment rate	9.1	10	9.3
No high school diploma	16 %	36 %	18 %
Proportion of visible minorities	33 %	54 %	49 %

A “ward” is one of the sections into which a city is divided for political/electoral purposes

Information obtained from Statistics Canada, National Household Survey (2011) [16]

Table 3 presents the program evaluation data. Approximately 21 (9.3 %) children were screened positive for a hearing deficit and referred to the pediatric audiology and otolaryngology clinic. Females were identified with hearing deficits more often than males, with 17 (81 %) of those being referred being female, *χ*^2^(1, *N* = 228) = 7.78, *p* < 0.01. Approximately 44 (19.3 %) participants required referral to a family physician for otoscopic abnormalities. Similarly, a greater proportion of females (44 participants, 66 %) were identified with otoscopic abnormalities, *χ*^2^(1, *N* = 228) = 4.24, *p* = 0.04. Reasons for referral are shown in Table 4. Occluding cerumen and mucoid/serous effusion were the most common causes for referral, accounting for 64.4 and 24.4 % of referrals, respectively.

**Table 3:** Program evaluation data

	Total	Male	Female
Number of participants	228	109 (48 %)	119 (52 %)
Audiological evaluation needed	21 (9.3 %)	4 (19 %)	17 (81 %)**
Family medicine referral needed	44 (19 %)	15 (34 %)	29 (66 %)*

*p < 0.05

**p < 0.01

**Table 4:** Reasons for referral

Clinical diagnoses	Number of referrals
Occluding cerumen	29 (64.4 %)
Effusion (mucoid or serous)	11 (24.4 %)
Acute otitis media	1 (2.2 %)
Tympanic perforation	2 (4.4 %)
Tympanosclerosis	1 (2.2 %)
Canal edema and discharge	1 (2.2 %)

## Discussion

This is an evaluation of a free student-run hearing screening program, “Kids2Hear”, offered to inner-city elementary schools. Our findings show that hearing deficits are common in young children, with about 9.3 % of the overall sample requiring referral for audiology assessment. In addition, 19 % of screened children required referral to a local family physician for otoscopic abnormalities. Occluding cerumen was the most common cause for referral while mucoid and serous effusions were the second most common cause. Furthermore, females were significantly more likely to be identified with either a hearing deficit or otoscopic abnormality. This association has not been previously reported in the literature and warrants further investigation.

Risk factors for middle ear effusions are numerous including host factors (age, race, anatomy, immunodeficiency, genetic predisposition) and environmental factors (second-hand smoke exposure, socioeconomic status, attending school/daycare) [10]. It is not surprising that middle ear effusions were among the most prevalent cause for referral as the Kids2Hear screenings took place during the fall and winter months when the incidence of otitis media with effusion is the highest in the northern hemisphere. These children were also attending school where they are more likely to come in contact with pathogens, and viral upper respiratory tract infections are a risk factor for Eustachian tube dysfunction and the subsequent development of otitis media [10].

In evaluating a screening program, several characteristics must be considered. The major goal of a screening program is to detect a disease at a stage when the treatment can be effective in reducing long-term complications [11]. The disease being screened for should be common with a readily available treatment. The screening test should be widely available, safe to administer, reasonable in cost, capable of detecting a high proportion of the disease, and lead to improved health outcomes [11]. The Kids2Hear program aims to identify hearing deficits and otoscopic abnormalities, a very common issue with readily available treatment, among young children at schools in lower socioeconomic areas. Though not specifically studied, one may assume that the children included in this cohort were less likely to have regular primary care or pediatric assessments during which significant amounts of cerumen impaction would be detected and managed. Identifying these hearing issues early and referring children for evaluation may prevent issues with speech and language acquisition as well as poor academic achievement [2]. The screening test is also safe to administer to children with minimal risks and only requires the use of an audiometer and otoscope, which are both widely available and capable of detecting a high proportion of hearing deficits and otoscopic abnormalities [12]. The screening is free for participants and the expenses of running the program are minimal. In addition, medical students have an opportunity to refine their clinical skills and learn the technique of otoscopic examination, which is important in pediatric assessment in many different medical fields [13]. Despite the fact that otolaryngologic problems are common, many medical training programs lack dedicated otolaryngologic training [13, 14]. This program enabled students to practise their skills with direct supervision and immediate feedback from experienced otolaryngologists and audiologists.

Although the overall concept of the Kids2Hear program is in accordance with the values that a screening program should embody, there were several limitations discovered in this study that limited our ability to assess the potential benefits of the screenings performed. The first is that this study includes a relatively small sample size and sample bias, as the schools participating in this program were not picked at random. Secondly, the accuracy of the audiometric methods used in the evaluation of screening for hearing loss should be discussed. The American Speech-Language-Hearing Association guidelines specify that hearing screening must be done with calibrated audiometers, in an environment with sufficiently low ambient noise (<49.5 dB SPL), and minimal distractions in the surrounding environment [15]. The Kids2Hear screening program used calibrated audiometers and kept environmental distractions to a minimum by appropriate placement of the screening areas, but ambient noise was not controlled for and in a school setting there can be quite a variation in ambient noise depending on the time of day and other ongoing activities. As well, although the 2011 American Academy of Audiology (AAA) guidelines state that the most widely preferred hearing screening procedure is the pure tone audiometric sweep test for children chronologically and developmentally aged three years and older, there is some current discussion around using otoacoustic emmisions (OAEs) instead for screening as a more promising option in children. The proponents for using OAEs, argue that OAE is an objective technique, in which findings are not influenced by as many listener variables (cognitive level, language skills, motor abilities, and distractions in the environment) that confound hearing screening with a behavioral technique (such as pure tone measurement) [15]. As seen in Table 1, the majority of children in this study come from homes where their primary language is not English. By using pure tone audiometry with instructions provided to the children in English, language comprehension may in itself decrease the accuracy of the screening outcomes.

Lastly, as this was a retrospective analysis, and the elementary students themselves were not enrolled in any form of research study, we were not provided with any baseline health or development information for these children (pre-existing hearing deficits, frequency of visits to their family physician, developmental delays, etc.), and we had no ability to follow-up on short or long-term outcomes of the children who were referred for further evaluation. As such, we were unable to correlate the results from the Kids2Hear screening evaluations with the official assessments in the audiology and otolaryngology clinics at HSC, unable to follow up with results and/or management that may have taken place with the local family physicians, and we were unable to track any treatment or interventions that may have taken place (ie. hearing aid fitting, surgery, speech-language consultations, etc.). We therefore do not have enough information to assess the potential effectiveness of this screening program at this time.

When combining the importance of detecting hearing deficits at an early age (to prevent long-term negative speech and language sequelae) [4], the fact that not all children are exposed to the same standard or amount of hearing screening during the newborn period [8], and that acquired or progressive hearing deficits may not be detected until later in childhood [7], one can highlight the importance of a screening program for hearing deficits either prior to entry into school (ideally), or during the first few years after starting school. This free medical student-run hearing screening program at the University of Toronto may very well be able to play an important role in filling this missing gap in preventative care and could eventually be a model for other communities; but as of yet, no conclusions can be drawn as to its effectiveness. Ongoing studies are needed to evaluate this program over the next few years, which will need to include follow-up of outcomes and measures of effectiveness,

## Conclusions

This is the first evaluation of a student-run free hearing screening program offered at inner-city schools in the literature. Our data demonstrates that hearing deficits and otoscopic abnormalities are common in young children, with 9.3 and 19 % of our overall sample requiring referral for further evaluation. Our findings also indicate that females were significantly more likely to be identified with hearing deficits or otoscopic abnormalities, which has not been demonstrated in the previous literature and could be an avenue for future research. As there was no follow-up on short or long-term outcomes of the children referred for additional evaluation, we currently do not have enough information to assess the potential benefits of this screening program. Additional research is needed to evaluate the Kids2Hear program over additional years to determine the program’s effectiveness.
